# A Case of Suppression of Urine in Which Relief Was Obtained from Puncturing the Bladder above the Pubis, but Which Afterwards Terminated Fatally; with an Account of the Appearances on Dissection; and Some Remarks on the Paracentesis of the Bladder

**Published:** 1790

**Authors:** Francis Turner

**Affiliations:** Surgeon at Yarmouth


### LONDON MEDICAL JOURNAL.

### I. *A Case of Suppreſſion of Urine in which Relief was obtained from punƐturing the Bladder above the Pubis, but which afterwards terminated fatally; with an Account of the Appearances on DiſſeƐtion: and ſome Remarks on the Paracenteſis of the Bladder. Communicated in a Letter to Dr.* Simmons, *F. R. S. by Mr.* Francis Turner, *Surgeon at Yarmouth.*

ON Monday, Auguſt 17th, 1789, a boy, three years old, was brought to me, labouring under a ſuppreſſion of urine, with which he had been ſeized on the preceding Thurſday. He had never had any ſymptom of ſuch a diſorder before; but a few days prior to the attack had complained of a pain in his bowels, which had been followed by a purging. From the time the ſuppreſſion had taken place he had voided only a few drops of urine, at different times, with great pain; and when I firſt ſaw him the bladder was diſtended conſiderably above the pubis.

I endea-

I endeavoured for ſome time, in a very gentle manner, to introduce a catheter, and afterwards a bougie; but proving equally unſucceſsful with both, I recommended a trial of a warm bath twice or thrice a day for an hour each time, and directed a tea-ſpoonful of caſtor oil to be adminiſtered every two hours.

On Tueſday the 18th, in the morning, the child having made no urine, and having paſſed a reſtleſs night from pain, I made another attempt to introduce the catheter, but with no better ſucceſs than before; I therefore deſired that the uſe of the caſtor oil and warm bath might be continued, and that a common oily clyſter might be injected.

The caſe proving thus obſtinate, I requeſted a medical friend to accompany me in my viſit to the patient in the evening; and the child being then ſtill in the ſame ſtate as before, we thought it neceſſary again to try the catheter, and both of us attempted to introduce it without ſucceſs: a bougie alſo was again ineffectually tried at this viſit.

The boy appeared now to be in more pain than before, and the bladder might eaſily be felt as high as the navel, conſiderably ſwelled and tenſe. The mode of treatment we had adopted was directed to be ſtill purſued, with the addition of an opiate at night.

Whilſt we were endeavouring to introduce the catheter, about a ſpoonful of urine eſcaped, and the catheter paſſed very well till it reached the neck of the bladder, or very near it; but when it had paſſed thus far, it appeared to be immoveably fixed.

On Wedneſday the 19th, Dr. Aikin, and three other gentlemen, ſurgeons in this town, met me in conſultation on the caſe, and as the patient had as yet voided no urine, we determined (after having again ineffectually tried the catheter) to puncture the bladder above the pubis; which operation I immediately performed, and near a pint of urine was drawn off. The bladder ſeeming to have ſlipped off the canula, the latter was withdrawn, and the puncture ſoon cloſed. The uſe of the caſtor oil, warm bath, and clyſters, was continued, and the patient had three grains of calomel given him by Dr. Aikin's direction. In the evening he was eaſy, but had ſlept much during the day. As he had had no ſtool, the caſtor oil was changed for the infuſion of ſena, of which he took two ſpoonfuls every two or three hours.

 Thurſday

Thurſday the 20th he had had a very good night, and was eaſy; the infuſion had operated well, but he had diſcharged no urine. The uſe of the warm bath was continued; and the infuſion was alſo directed to be repeated, but with leſs frequency.

Friday the 21ſt he was very uneaſy, had made no urine, and the bladder was as much diſtended as on the Wedneſday. It was therefore, in conſultation, again determined to try the catheter, and that not ſucceeding, I again punctured the bladder above the pubis. Nearly the ſame quantity of urine was evacuated as before, and the canula was left in the bladder. Four grains of calomel and one of antim. tartariſat. were directed to be divided into four doſes, and one of them to be given every four hours. In the evening he was eaſy; and the urine paſſed very freely through the canula.

Saturday the 22d he was very eaſy, and had had ſeveral looſe ſtools in the night; on which account it was agreed to diſcontinue the uſe of his medicines, and to let him have mild nouriſhment; but on the 24th, as he was again coſtive, we thought it right to repeat the infuſion of ſena, and after taking it he had ſeveral looſe ſtools.

On

On the 26th he had made no urine by the penis, but it was freely evacuated through the canula; the abdomen was considerably leſs tenſe, and his bowels were in a lax ſtate.

I now introduced a bougie as far as I could, and left it in the urethra: I did ſo again in the evening, and it ſeemed then to paſs the whole way; but of this I could not be certain, as it did not move ſo freely as a bougie uſually does when it enters the bladder.

Thurſday the 27th the nurſe told me the patient had made ſome urine, and I was much pleaſed to find ſome between the glans penis and prepuce, the extremity of which was very tight. In the courſe of this day he made urine as freely as he ever did, but appeared to be in pain at the time. His purging continued; his pulſe was quick; and as he was reſtleſs the opiate was repeated at night.

Friday the 28th he voided his urine freely; the purging continued; and the opiate was repeated.

Saturday the 29th I withdrew the canula; his pulſe was ſtill quick, and his purging continued.

Sunday, September 6th, his pulſe continued quick; his purging was not abated; and he had very little appetite. The urine had come through both penis and puncture till yesterday, when it all came by the penis. About this time the scrotum beginning to swell, I applied to it a common poultice; the swelling and hardness increased, a suppuration took place, and on Wednesday, September 16th, it burst, and discharged some pus. His appetite was now rather better, and his fever less; but his purging still continued.

Sunday, September 20th, the scrotum was completely healed, and reduced to its natural size. It was at this period of the case, Sir, that I communicated an account of it to you. He then, and indeed for some time after, voided his urine very freely, and without pain, so that I had the greatest hope of his recovery. But about the latter end of October his appetite grew indifferent, and he had frequent vomitings. The abdomen, in the mean time, became enlarged, and a considerable hardness might plainly be felt above the os pubis.

He continued nearly in the same state till November 30th, on which day he died.

From the time of his urine coming freely after the punctures, till his death, he had no complaint in making it.

I opened

I opened the body within a few hours after his death, and the gentlemen who were with me on the former occasions did me the favour of attending on this.

The body was very much emaciated, and the abdomen very large; a hardness was to be felt, extending higher than the navel, and reaching as far as the os ilium on each side.

Upon turning back the integuments and abdominal muscles there appeared a tumour every where smooth, shining, and hard. I could pass my fingers behind this tumour easily, rather beyond the upper part of the os sacrum.

In the centre of the tumour, just above the os pubis, was the bladder, attached, in a flat manner, in its whole posterior part, to the tumour. Its anterior part was convex, but not so much so as in a natural state. It contained very little urine, and was indeed so much contracted as to be incapable of holding more than three ounces. Upon pressing the bladder, urine came freely through the penis.

The tumour filled the pelvis completely, and extended upwards, as hath been mentioned. On being dissected out, it appeared to be much larger than an infant's head, and nearly of the same shape. Upon cutting it through it was found to contain, in different cavities, about four ounces of a slimy discoloured fluid. In every other part it was of a very hard substance. It adhered to the rectum, and had no communication with the cavity of the bladder. The viscera of the abdomen were all sound.

This disease was absolutely incurable; but is, I think, no objection whatever to the place where the punctures were made, as every intention was answered by them; and if the disease had been of a curable nature, the boy would have had a great chance for recovery, as the bladder did not appear in the least injured from the canula.

In suppressions of urine, where perforation of the bladder is necessary, surgeons are not decided as to the most eligible part for making the perforation; some recommending the puncture to be made above the pubis, others in the perinæum, and some through the rectum. The late Mr. Samuel Sharpe, whose opinion has deservedly great weight in every thing that relates to the operative part of surgery, was in favour of the puncture above the pubis; Mr. Bell, to whom we are indebted for an excellent system of surgery, inclines to that in the perinæum; and there are others, particularly some French surgeons

ſurgeons of eminence, who recommend the perforation of the bladder through the rectum.

If I may venture my opinion on this ſubject, I muſt confeſs I agree with Mr. Sharpe, more eſpecially where the ſuppreſſion depends on inflammation at the neck of the bladder and proſtate gland, as in ſuch caſes the diſtance from the ſeat of the inflammation muſt give it the preference.

The adheſion of the calculous matter to the part of the canula in the bladder, and the pain that attends its removal after a certain time, together with the difficulty of again introducing it, are arguments made uſe of againſt the puncture above the pubis: but theſe objections, if my obſervations are well founded, are of no moment; for an inflammation comes on at the edges of the puncture, which in a very ſhort time connects the external edges of the wound in the bladder with the internal edges of the wound in the abdominal muſcles. This connexion takes place in a very ſhort time, as was proved to me ſome years ago by a diſſection I then made.

A ſea-faring man, between forty and fifty years of age, was brought aſhore at this place with a total ſuppreſſion of urine from ſtrictures in in the urethra. In this cafe, perforation of the bladder becoming abfolutely neceffary, a puncture was made above the pubis. The man died a few days after the operation, and before a bougie could be made to reach the bladder. On diffection, the adhefion of the mufcular coat of the bladder to the tranfverfalis mufcle of the abdomen, all round the puncture, was found to be very ftrong.

In the cafe, which is more particularly the fubject of the prefent paper, the boy pulled out the canula on the Saturday afternoon, and the nurfe then replaced it. After that, by the patient's crying and ftraining, it was forced out feveral times. This fometimes happened when I was prefent. After well oiling the puncture and canula, I flowly, and in a little time, introduced it again; there is, therefore, no hazard in taking out the canula after it has been in a few days.

The trocar I employed was fuch as I ufe in the palliative method of treating the hydrocele: but I think the trocar fhould be curved; in that cafe, if the canula is too long, the fmooth part of it will prefs againft the pofterior part of the bladder, and occafion much lefs harm than the extremity of the ftraight canula, which may may ulcerate the bladder, and eaſily make its way into the rectum. The trocar ſhould alſo have rings, to which tapes may be affixed and faſtened round the body.

The trocar for the adult ſhould be of the ſize of that uſed in the paracenteſis of the abdomen, and ſhould not exceed two inches and a half in length, including a proper curve: that for a child may be ſuch a one as is uſed in tapping the hydrocele; and this alſo ſhould be properly curved.

*Yarmouth,*
December 1, 1789.

